# Advancing biomedical applications: an in-depth analysis of silver nanoparticles in antimicrobial, anticancer, and wound healing roles

**DOI:** 10.3389/fphar.2024.1438227

**Published:** 2024-08-08

**Authors:** Himanshu Jangid, Sudhakar Singh, Piyush Kashyap, Avtar Singh, Gaurav Kumar

**Affiliations:** ^1^ School of Bioengineering and Biosciences, Lovely Professional University, Jalandhar, Punjab, India; ^2^ School of Agriculture, Lovely Professional University, Jalandhar, Punjab, India; ^3^ School of Electrical Engineering and Computing (SoEEC), Adama Science and Technology University (AS-TU), Adama, Ethiopia

**Keywords:** silver nanoparticles, antimicrobial resistance, cancer nano therapy, wound healing innovations, sustainable synthesis methods and bibliometric trend analysis

## Abstract

**Introduction:** Silver nanoparticles (AgNPs) have gained significant attention in biomedical applications due to their unique physicochemical properties. This review focuses on the roles of AgNPs in antimicrobial activity, anticancer therapy, and wound healing, highlighting their potential to address critical health challenges.

**Methods:** A bibliometric analysis was conducted using publications from the Scopus database, covering research from 2002 to 2024. The study included keyword frequency, citation patterns, and authorship networks. Data was curated with Zotero and analyzed using Bibliometrix R and VOSviewer for network visualizations.

**Results:** The study revealed an increasing trend in research on AgNPs, particularly in antimicrobial applications, leading to 8,668 publications. Anticancer and wound healing applications followed, with significant contributions from India and China. The analysis showed a growing focus on “green synthesis” methods, highlighting a shift towards sustainable production. Key findings indicated the effectiveness of AgNPs in combating multidrug-resistant bacteria, inducing apoptosis in cancer cells, and promoting tissue regeneration in wound healing.

**Discussion:** The widespread research and applications of AgNPs underscore their versatility in medical interventions. The study emphasizes the need for sustainable synthesis methods and highlights the potential risks, such as long-term toxicity and environmental impacts. Future research should focus on optimizing AgNP formulations for clinical use and further understanding their mechanisms of action.

**Conclusion:** AgNPs play a pivotal role in modern medicine, particularly in addressing antimicrobial resistance, cancer treatment, and wound management. Ongoing research and international collaboration are crucial for advancing the safe and effective use of AgNPs in healthcare.

## 1 Introduction

Silver nanoparticles (AgNPs) have emerged as a captivating and highly versatile class of nanomaterials, drawing significant attention from the scientific community across diverse fields (Akter et al., 2018; Xu et al., 2020). Their unique physicochemical properties, distinct from bulk silver and other traditional materials, arise from their nanoscale dimensions (Zhang et al., 2016; Galatage et al., 2021). In recent years, AgNPs have garnered immense interest in biomedical research, particularly for their remarkable applications in antibacterial, anti-cancer, and wound healing therapies (Hasan et al., 2022; Hou et al., 2023). The exceptional properties of AgNPs stem from their remarkably large surface area-to-volume ratio, which confers upon them exceptional reactivity, making them ideal for various surface-related applications (Rani et al., 2020; Bruna et al., 2021). This heightened reactivity is crucial for their interactions with biological systems and enhances their efficacy in biomedical applications. One of the most compelling attributes of AgNPs is their potent antimicrobial activity. This activity is attributed to the controlled release of silver ions, which disrupt microbial cell membranes and internal processes, leading to the destruction of a broad spectrum of pathogens, including bacteria, viruses, and fungi (Yin et al., 2020). Notably, AgNPs have shown promise in combating antibiotic-resistant bacterial strains like Methicillin-resistant *Staphylococcus aureus* (MRSA) and multidrug-resistant Enterobacteriaceae, offering a potential solution to the growing global threat of antibiotic resistance (Dove et al., 2023; dos Santos et al., 2021; More et al., 2023). Beyond their antimicrobial prowess, AgNPs exhibit tunable optical and electronic properties that can be finely adjusted by manipulating their size, shape, and surface chemistry (Dikshit et al., 2021). This unique feature opens up a wide array of possibilities for their utilization in various fields, including sensing, imaging, and drug delivery. For instance, AgNPs can be engineered to act as highly sensitive sensors for detecting specific biomolecules or pathogens, providing valuable tools for diagnostics and disease monitoring. Moreover, their ability to absorb and scatter light in a controlled manner makes them suitable for imaging applications, potentially enabling the visualization of biological processes at the cellular and molecular levels. In the realm of biomedical research, AgNPs have demonstrated immense potential in combating infectious diseases, and cancer, and facilitating wound healing. As antibiotic resistance continues to pose a significant challenge to global health, AgNPs offer an alternative and complementary therapeutic approach. They are increasingly being integrated into medical devices, wound dressings, and topical formulations to prevent and treat infections (Qing et al., 2018; Paladini and Pollini, 2019). The incorporation of AgNPs into wound dressings, for example, has been shown to reduce infection rates and accelerate the healing process of chronic wounds such as diabetic ulcers and burns, offering hope for patients with difficult-to-treat wounds.

Furthermore, AgNPs have shown remarkable promise in cancer research. Their ability to selectively induce cytotoxicity in cancer cells while sparing healthy tissue makes them attractive candidates for targeted anti-cancer therapies (Zielinska et al., 2017). Researchers have extensively explored AgNPs as carriers for drug delivery, aiming to precisely deliver chemotherapeutic agents to malignant cells, thereby minimizing systemic side effects and enhancing the efficacy of cancer treatments (Gomes et al., 2021; Xu et al., 2023). The potential of AgNPs in revolutionizing cancer therapy is a rapidly evolving area of research with significant implications for the future of oncology. In addition to their antibacterial and anti-cancer properties, AgNPs play a crucial role in wound healing. Their antimicrobial properties create a favorable environment for tissue regeneration and wound closure, making them valuable components in advanced wound care materials (Krishnan et al., 2020; Rybka et al., 2023). The incorporation of AgNPs into wound dressings and hydrogels has shown promising results in accelerating healing in burn injuries, chronic ulcers, and other persistent wounds, offering a ray of hope for patients suffering from debilitating and slow-healing wounds.

The following are the objectives of the studies:

➢ To Conduct a Comprehensive Bibliometric Analysis: The primary objective of this research is to perform a comprehensive bibliometric analysis of the existing body of literature related to silver nanoparticles (AgNPs) in biomedical applications, with a specific focus on antibacterial, anti-cancer, and wound healing properties.
➢ To Identify Pivotal Trends: This research aims to identify and analyze pivotal trends in AgNP research, including publication trends, authorship patterns, citation networks, and keyword usage.
➢ To Recognize Influential Authors and Studies: Another objective is to recognize influential authors, research groups, and studies in the field of AgNP applications for antibacterial, anti-cancer, and wound healing purposes.
➢ To Delineate Emerging Research Trajectories: The research seeks to delineate emerging research trajectories within the realm of AgNPs, shedding light on evolving areas of interest and potential future directions in biomedical applications.

## 2 Literature review

Silver nanoparticles (AgNPs) have emerged as pioneers in nanotechnology applications owing to their distinctive attributes. Their utility transcends conventional boundaries, offering significant progress in antimicrobial treatments, anticancer therapies, and wound healing processes (Firdhouse and Lalitha, 2015; Bruna et al., 2021). The adaptability of AgNPs, characterized by their size-dependent physical and chemical properties, has spurred innovative approaches in medical science and biotechnology (Chung et al., 2016).

In antimicrobial applications, AgNPs have displayed exceptional efficacy against a broad spectrum of pathogenic microorganisms, including bacteria resistant to traditional antibiotics. This has paved the way for the development of more potent and less resistance-prone antimicrobial agents (More et al., 2023; Kim et al., 2024). Additionally, the eco-friendly synthesis of AgNPs has underscored a sustainable approach to harnessing their antimicrobial potential, minimizing environmental impact while maximizing therapeutic benefits (Osman et al., 2024). In the realm of anticancer research, AgNPs have exhibited promising capabilities in targeting and inhibiting tumor growth. Their versatility in being tailored for specific drug delivery systems has augmented the effectiveness of anticancer drugs, providing a focused approach to cancer treatment (Onugwu et al., 2022). The distinct properties of AgNPs, such as their surface modification and bioconjugation potential, have facilitated the creation of innovative anticancer therapies with reduced side effects and improved patient outcomes. Moreover, the applications of AgNPs in wound healing have yielded remarkable outcomes (Takáč et al., 2023). Their antimicrobial attributes, coupled with their capacity to stimulate tissue regeneration, have rendered AgNPs invaluable in the development of advanced wound dressings and healing agents. Incorporating AgNPs in wound care not only prevents infections but also expedites the healing process, enhancing recovery times and patient care (Aldakheel et al., 2023).

The subsequent Tables 1–3 succinctly outline significant breakthroughs in the utilization of silver nanoparticles across these critical research domains. Table 1: Key Breakthroughs in Silver Nanoparticles Antimicrobial Research Area Table 1 lists some of the key results in this field of research on silver nanoparticle antimicrobial science. It covers diverse synthesis methods, from chemical and green to biological syntheses, and the respective mechanisms associated with them for combating microorganisms. For instance, the AgNPs synthesized with the extract from green tea showed high activity against several bacterial strains while being biocompatible with human cells. This table underlines the multi-mechanistic properties of AgNPs, which include penetration and damage to bacterial cell walls, increase in membrane permeability, and generation of reactive oxygen species. There are, however, gaps regarding optimal dosing and long-term environmental effects; thus, research should be channeled toward these areas to maximize antimicrobial effectiveness and safety. Table 2: Key Breakthroughs in Silver Nanoparticle’s Anti-Cancer Research Domain Table 2 presents the development of the anticancer uses of AgNPs. These are antitumor activity of papaya leaf extracts mediated AgNPs against prostate cancer through pathways including cell arrest and apoptotic induction. The table suggests a need for strong *in vivo* testing and clinical trials to verify the findings in terms of efficacy and safety. In addition, a description of the specific cellular targets and an understanding of the molecular mechanisms implicated will be necessary to progress from these promising results to clinical application. Table 3: Key Milestones in the Domain of Wound Healing Research on Silver Nanoparticle Table 3 illustrates certain wound healing applications studies of AgNPs and their mechanisms of work to manifest dual features; AgNPs promote tissue growth on one side and hinder infections on the other side. For instance, AgNPs-based hydrogel dressings have improved diabetic rat healing by downregulating oxidative stress and inflammation pathways. Also, the most critical contribution given by biocompatible and eco-friendly synthesis methods includes using natural extracts for synthesizing AgNPs. Future perspectives in research should be optimization of these synthesis techniques for increased production efficiency and evaluation of the long-term effects of AgNPs in clinical applications for wound healing.

**TABLE 1:** Key breakthrough in silver nanoparticles antimicrobial research domain.

Key findings	Synthesis method	Antimicrobial mechanism	Research gap	Future directions	Reference
AgNPs exhibit multiple and simultaneous mechanisms of action against Gram-negative and Gram-positive bacteria, including multidrug-resistant strains	-	Multiple mechanisms, including penetration and disruption of bacterial cell walls	Determining the optimal dosages to minimize cytotoxic effects while maximizing antimicrobial efficiency	Synergistic use with antibiotics to enhance efficacy and reduce dosage	Bruna et al. (2021)
Green synthesis of AgNPs is highlighted as an eco-friendly alternative, offering novel antimicrobial therapies against resistant pathogens	Green synthesis using biological agents, plants, or microbial agents	Not specified in the abstract. Likely involves interaction with microbial cell membranes leading to cell death	Comprehensive assessment of the long-term environmental and health impacts of green-synthesized AgNPs	Advancements in green synthesis methods to increase efficiency and reduce production costs	Roy et al. (2023)
Bacterial-mediated AgNPs showed significant antimicrobial potential against MRSA and several MDR bacteria	Bacterial-mediated synthesis from strains like *Escherichia coli* and *Brevundimonas diminuta*	The mechanism involves disruption of microbial cell membranes and inhibition of growth	Optimization of bacterial strains and conditions for maximum AgNP production	Exploring the use of different bacterial strains and optimizing conditions for enhanced antimicrobial efficacy	Saeed et al. (2020)
Green synthesized AgNPs using *Pseudoduganella eburnea* showed potent antimicrobial activity against drug-resistant pathogens	Green synthesis using *Pseudoduganella eburnea*	Structural changes and destruction of membrane integrity in bacteria like *S. aureus* and *P. aeruginosa*	Further investigation into the antibacterial mechanisms and potential resistance development	Development of AgNP-based antimicrobial treatments for therapeutic applications	Huq (2020)
AgNPs penetrate bacterial cell walls, changing the structure of cell membranes and resulting in cell death, with applications in dentistry noted	-	Penetration of cell walls, increased membrane permeability, production of reactive oxygen species, and disruption of DNA.	Investigating the long-term effects of AgNP exposure in dental applications and potential resistance in oral microbes	Enhancing the incorporation of AgNPs in various dental materials for improved antimicrobial properties	Yin et al. (2020)
Developed AgNPs with pH-induced surface charge switchable properties, showing promise in decreasing cytotoxicity to healthy cells while increasing antibacterial and antibiofilm efficiency	Chemical synthesis with carboxyl betaine groups for pH responsive properties	pH responsive charge switching enables adherence to bacterial surfaces and penetration into biofilms based on electrostatic attraction	Assessing the long-term biocompatibility and potential environmental impact of these novel AgNPs	Exploration of these AgNPs in various biomedical applications, particularly for targeting biofilm-associated infections	Qiao et al. (2019)
Morphological dependence of antimicrobial activity of AgNPs was observed, with spherical AgNPs showing greater efficacy	Chemical synthesis with controlled shape and size parameters	The antimicrobial activity varies with shape due to differences in Ag ion release rates	Understanding the precise mechanisms by which nanoparticle shape influences antimicrobial efficacy	Tailoring the shape and size of AgNPs for specific antimicrobial applications, enhancing effectiveness against MDR strains	Cheon et al. (2019)
Demonstrated the biogenic synthesis of AgNPs using green tea extract, highlighting their potent antimicrobial activity and biocompatibility with human keratinocyte cells	Biogenic synthesis using green tea extract as a reducing and stabilizing agent	Antibacterial activity against multiple bacterial strains without significant toxicity to human cells	Comparative analysis of different biogenic sources for AgNP synthesis to optimize antimicrobial activity and biocompatibility	Exploration of biomedical applications where biogenically synthesized AgNPs can be effectively utilized	Rolim et al. (2019)
Biologically synthesized AgNPs using *Sphingobium* sp. MAH-11 showed strong anti-microbial activity against drug-resistant pathogenic microorganisms	Biological synthesis using *Sphingobium* sp. MAH-11	Disruption of microbial cell membranes and integrity, particularly in *E. coli* and *S. aureus*	Detailed investigation into the long-term effects and potential resistance mechanisms to biologically synthesized AgNPs	Further exploration of eco-friendly synthesis methods and the application of AgNPs in therapeutic settings	Akter and Huq (2020)
AgNPs at biocompatible dosages synergistically increased bacterial susceptibility to antibiotics, highlighting a potential strategy against antimicrobial resistance	Chemical synthesis from silver nitrate with glucose reduction	Synergistic effect with antibiotics, enhancing bacterial growth inhibition even at non-cytotoxic concentrations	Further *in vivo* testing to validate the efficacy and safety of AgNP-antibiotic combinations for clinical use	Development of novel antimicrobial strategies combining AgNPs with antibiotics to combat resistant bacterial infections	Ipe et al. (2020)
Mycologically synthesized AgNPs exhibited enhanced antibacterial capability against MDR pathogens	Biological synthesis using fungal strains, particularly Penicillium notatum	Not specified in abstract. Likely involves disruption of bacterial cell membranes and inhibition of cellular functions	Exploration of the full spectrum of fungal species capable of synthesizing AgNPs with superior antimicrobial properties	Utilizing a wider range of fungal species for the eco-friendly production of AgNPs with targeted antimicrobial activities	Hayat et al. (2023)

**TABLE 2:** Key breakthroughs in silver Nanoparticle’s anti-cancer research domain.

Key findings	Synthesis method	Anticancer mechanism	Research gap	Future directions	Reference
AgNPs control growth rate of human liver and breast cancer cells	Trisodium citrate salt was employed as a reducing agent	Induction of ROS, apoptosis via gene expression regulation	Depth of *in vivo* studies and long-term effects	Extensive *in vivo* testing and clinical trials	Al-Khedhairy and Wahab (2022)
Demonstrated antitumoral mechanisms of biogenic AgNPs in bladder cancer	Synthesized from *Fusarium* sp.	Apoptosis induction, cell migration and proliferation inhibition	Molecular mechanisms towards bladder cancer	Exploration of AgNPs in other cancer models and clinical evaluation	Ferreira et al. (2020)
AgNPs with papaya leaf extract show anti-cancer properties against prostate cancer	Biosynthesis with papaya leaf extract	Cell cycle arrest, ROS production, apoptosis induction	Specific cellular targets for nanoparticles’ action	Identification of specific molecular targets and clinical applicability	Singh et al. (2021)
Exploration of AgNPs as potential therapeutics in cancer biology	Not specified	Various anticancer features including targeted therapy	Clinical utilization hurdles and comprehensive mechanisms	Clinical trials to determine efficacy and safety in humans	Miranda et al. (2022)
AgNPs show cytotoxicity to TNBC cells without affecting non-malignant cells	Not detailed	TNBC-specific cytotoxicity via nanoparticle formulation	Detailed mechanism of TNBC cell specificity	Development for safe and specific TNBC treatments	Swanner et al. (2019)
Evaluated anti-cancer activity of AgNPs synthesized from *Zingiber officinale* leaf	Green synthesis with *Zingiber officinahhle* leaf extract	Inhibition of cell viability and proliferation in pancreatic cancer cells	Clinical trials for human application	Clinical validation and optimization for pancreatic cancer treatment	Wang et al. (2021)
Developed BSA-coated AgNPs for multimodal therapy of skin cancer	Single-step reduction process	Cytocidal effects, anti-angiogenic effects, and light-to-heat conversion for photothermal therapy	Comparative efficacy and safety studies	Further studies to explore its applicability in other cancer types	Kim et al. (2021)
Reviews cytotoxic effects of green-synthesized AgNPs in cancer models	Green synthesis following green chemistry recommendations	Size, shape, capping, and SPR profile dependent cytotoxic effects	Standardization and full characterization	Additional studies for a comprehensive understanding and clinical applications	Morais et al. (2020)
Green-synthesized AgNPs induce apoptotic cell death in breast cancer cells	Green synthesis from Fagonia indica extract	Activation of caspases, ROS generation, apoptotic cell death	Molecular mechanism of apoptosis induction	Detailed investigation into the mechanism and potential for clinical trials	Ullah et al. (2020)

**TABLE 3:** Key breakthroughs in silver nanoparticles wound healing research domain.

Key findings	Synthesis method	Wound healing mechanism	Research gap	Future directions	Reference
Developed ultrafine AgNPs (∼2 nm) using γ-cyclodextrin MOFs, enhancing stability and dispersibility in aqueous media	Template-guided synthesis using γ-cyclodextrin MOFs	Enhanced antibacterial effect and promoted wound healing via improved stability and dispersibility	Limited *in vivo* data on long-term biocompatibility and toxicity	Further *in vivo* studies to assess long-term effects and explore clinical applications	Shakya et al. (2019)
Developed a novel wound dressing from natural materials and AgNPs, showing antioxidant and anti-inflammatory properties for *in-vivo* wound healing	Electrospinning of Polygalacturonic and Hyaluronic acid embedded with AgNPs	Antioxidant and anti-inflammatory properties accelerate wound healing	Assessment of the long-term effects and the integration process of the nanofiber mat in various wound types	Exploration of the scalability of the electrospinning process for commercial production	El-Aassar et al. (2020)
Accelerated wound healing in diabetic rabbits using a chitosan-PEG hydrogel impregnated with AgNPs, highlighting sustained release and antimicrobial properties	Reduction of silver nitrate with PEG and chitosan solution	Sustained release of AgNPs providing antimicrobial and improved wound healing capabilities	Effectiveness in non-diabetic wound models and comparison with other nanomaterials	Comparative studies on the effectiveness of this hydrogel with other nanomaterials in various wound types	Masood et al. (2019)
Utilized curcumin-cyclodextrin complex for green synthesis of AgNPs, loaded into bacterial cellulose hydrogels, offering antimicrobial activity and promoting wound healing	Green synthesis using curcumin and hydroxypropyl-β-cyclodextrin complex	Antimicrobial activity against common wound-infecting pathogens and promotion of wound healing	Investigation into the release kinetics of AgNPs from the hydrogel and its effect on healing efficiency	Development of controlled release systems for AgNPs to optimize healing efficiency	Gupta et al. (2020)
Developed G-AgNPs reduced with ethylcellulose, demonstrating enhanced wound healing properties in a rat model, supporting rapid healing	Reduction with ethylcellulose and incorporation into an oil-in-water cream base	Enhanced formation of granulation tissue and proliferation of epithelial tissue	The impact of different concentrations and formulations on various skin types and wound conditions	Optimization of formulation for different skin types and wound conditions for broader applicability	Abdellatif et al. (2023)
Created Chi/Ag-NPs showing significant antibacterial, antibiofilm, antifungal, antioxidant, and wound healing activities	Chemical reduction of silver ions in the presence of chitosan	Accelerates wound healing process by enhancing fibroblast migration and reducing microbial load	Exploration of the specific mechanisms through which Chi/Ag-NPs influence cellular behavior and wound healing	Further studies on the molecular mechanisms involved and the potential for scaling up production for clinical use	Shehabeldine et al. (2022)
Demonstrated superior wound healing acceleration and antimicrobial activity using biogenic silver nanoparticles (BSNP)	Biogenic synthesis using plant extracts	Enhanced fibroblast migration and reduced microbial load at wound sites	Comprehensive understanding of the interaction between BSNP and cellular components during healing	Detailed investigation into the cellular and molecular mechanisms of BSNP-enhanced wound healing	Giri et al. (2019)
Explored the combination of gallocatechin and silver nanoparticles for wound healing in diabetic rats, highlighting the suppression of oxidative stress and inflammation	Green synthesis utilizing gallocatechin for the reduction of silver ions	Modulated oxidative stress and inflammation via the Nrf2/HO-1 and TLR4/NF-κB pathways, improving collagen synthesis and wound healing	Efficacy and safety in human subjects, especially diabetic patients with chronic wounds	Clinical trials to validate efficacy and safety in humans, particularly in diabetic wound care	Vendidandala et al. (2021)
Demonstrated effective wound healing using AgNPs synthesized in the solid state, highlighting the potential of AgNPs-coated cotton fabrics	Solid-state synthesis using dextran as a reducing and stabilizing agent	Promoted antimicrobial properties and enhanced wound healing through effective bacterial suppression	Understanding the interaction between AgNPs and the cotton fabric in promoting wound healing	Investigation into long-term stability of AgNPs on fabrics and their efficacy in real-world applications	Elnaggar et al. (2021)
Developed chitosan composite sponge dressings loaded with iturin-AgNPs, showing enhanced antibacterial activity and promoting wound healing	Biosynthesis using iturin for the production of AgNPs	Enhanced re-epithelialization, collagen formation, and antibacterial activity leading to improved wound healing	Elucidation of the specific role of iturin in the biosynthesis process and its impact on the biological properties of AgNPs	Further exploration of biosynthetic approaches to optimize wound healing efficacy and reduce potential toxicity	Zhou et al. (2021)
Reviewed the potential of green-synthesized AgNPs loaded in Polyacrylamide hydrogel for wound healing, highlighting the eco-friendly approach	Green synthesis using natural sources like plant extracts for AgNP production	Accelerated wound healing process due to the antimicrobial properties of AgNPs and the supportive hydrogel matrix	Comprehensive evaluation of green synthesis methods for their efficiency, scalability, and impact on nanoparticle properties	Development and comparative analysis of various green synthesis methods to optimize AgNP characteristics for wound healing	Aldakheel et al. (2023)
Explored the antibacterial and wound healing properties of AgNPs biosynthesized by cyanobacterium *Phormidium* sp., showing topical effectiveness	Biosynthesis using cyanobacterium *Phormidium* sp.	Antimicrobial activity against MRSA and promotion of wound healing processing and quality	The impact of various cyanobacterial species on the synthesis and properties of AgNPs and their specific wound healing capabilities	Investigation into other cyanobacterial species for the biosynthesis of AgNPs with tailored properties for specific wound healing applications	Younis et al. (2021)
Investigated the effects of AgNP on wound healing using a zebrafish fin regeneration model, revealing potential toxicity at certain concentrations	Not specified	Impaired fin regeneration due to AgNP exposure, highlighting potential toxicity concerns	Detailed understanding of the toxicity mechanisms of AgNPs at the cellular and molecular level during wound healing	Development of safer AgNP formulations and concentrations that maximize wound healing while minimizing toxicity	Pang et al. (2020)

## 3 Methodology

### 3.1 Data collection

#### 3.1.1 Selection of databases

Scopus was selected as the main database for this bibliometric analysis because of its extensive coverage, multidisciplinary focus, detailed metadata, ease of use, and rigorous quality checks. Scopus offers access to a diverse array of scholarly articles, making it well-suited for our interdisciplinary examination of the applications of silver nanoparticles in antibacterial, anti-cancer, and wound healing contexts. It's timely updates and trustworthy data outlets guarantee the accurate tracking of the most recent research trends (Schotten et al., 2018; Pranckutė, 2021).

#### 3.1.2 Search strategy

A systematic search on Scopus was conducted, focusing on research articles written in English. The search utilized the keywords ‘Silver nanoparticle’ and ‘Anticancer activity’ and ‘Antimicrobial activity’ and ‘Wound healing.’ This search strategy aimed to narrow down the dataset to articles directly related to silver nanoparticles and their applications in antibacterial, anti-cancer, and wound healing contexts, with a further limitation to research articles (Qamer et al., 2021; Hak et al., 2022).

#### 3.1.3 Inclusion and exclusion criteria

Inclusion criteria for this bibliometric analysis encompassed research articles written in the English language. The chosen timeframe (2002–2024) ensured that the analysis included contemporary research in the field of silver nanoparticles (AgNPs) in biomedical applications. Articles were included if they directly pertained to AgNPs and their utilization in antibacterial, anti-cancer, or wound healing applications, as indicated by the presence of relevant keywords such as “silver nanoparticle” “anticancer activity” “antimicrobial activity” “wound healing” in the article content. Conversely, exclusion criteria consisted of non-English publications. Irrelevant articles that did not meet the defined keyword criteria or lacked relevance to the biomedical applications of AgNPs were excluded from the analysis. This stringent approach ensured the selection of a high-quality dataset directly aligned with the research objectives of this bibliometric study (Hinojo Lucena et al., 2019; Linnenluecke et al., 2019).

#### 3.1.4 Data extraction

A systematic method was utilized to gather crucial metadata for each chosen article. This data encompassed details such as the article’s title, authors, publication date, abstract, keywords, journal source, and citation count. The extracted data formed the basis for the subsequent bibliometric analysis (Mengist et al., 2020).

### 3.2 Bibliometric indicators

#### 3.2.1 Publication trends

This constructed a chronological depiction of research publications on AgNPs to identify trends in publication patterns over time. This timeline graphically represented the yearly distribution of articles across the designated research areas (Oliveira et al., 2019).

#### 3.2.2 Authorship patterns

To pinpoint key figures in the field, we thoroughly investigated patterns of authorship. We identified prolific authors, research collectives, and affiliations. Co-authorship networks were scrutinized to uncover patterns of collaboration and influential clusters of research (Carchiolo et al., 2022).

#### 3.2.3 Citation analysis

Citation analysis was performed to assess the impact of individual articles and their influence on subsequent research. Additionally, it built citation networks to visualize the interconnections among research within the biomedical domain of AgNP (Trujillo and Long, 2018).

#### 3.2.4 Keyword analysis

A comprehensive keyword analysis was undertaken to assess keyword frequency and significance. This aimed to identify frequently used keywords and emerging keywords indicative of evolving research areas (Chen and Xiao, 2016).

### 3.3 Data analysis

#### 3.3.1 Descriptive statistics

Fundamental descriptive statistics, encompassing metrics like mean, median, and standard deviation, were computed. These metrics were utilized to analyze publication trends, authorship structures, citation tallies, and keyword occurrences, furnishing a quantitative comprehension of the data (Cooksey, 2020).

#### 3.3.2 Visualization

Vosviewer, R-studio, and MS Excel serve as tools for visualizing data, enabling the creation of charts, graphs, and network diagrams to improve the presentation of results. Visual depictions were utilized to communicate intricate relationships and trends discovered during the analysis (Aria and Cuccurullo, 2017; Derviş, 2020; Guleria and Kaur, 2021; Bhat et al., 2023).

## 4 Results and discussion

**FIGURE 1:**
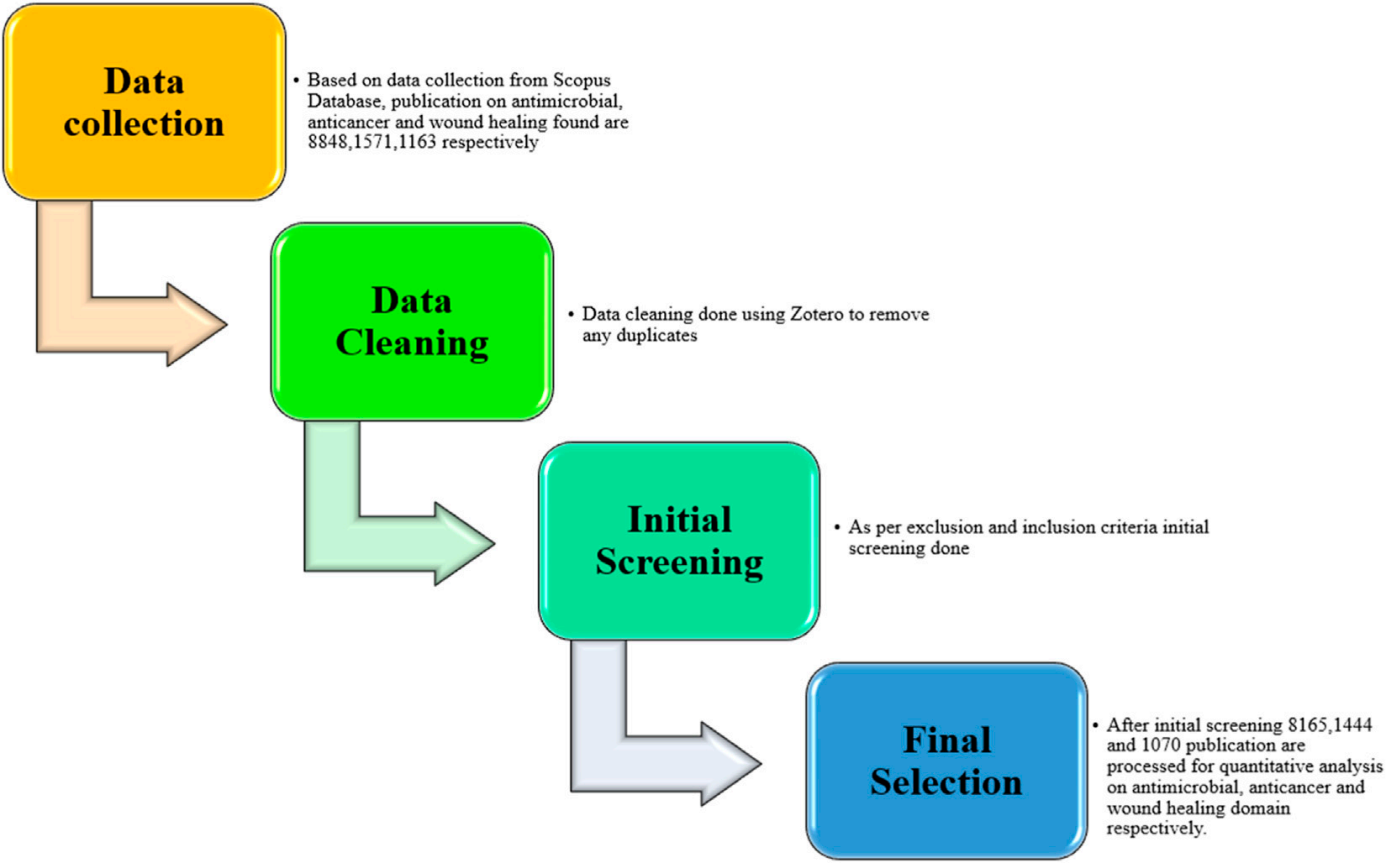
PRISMA flow diagram for bibliometric analysis of silver nanoparticles.

A bibliometric analysis was conducted using publications sourced from the Scopus database on 5 March 2024, focusing on interdisciplinary research concerning silver nanoparticles (AgNPs), particularly their applications in antimicrobial activity, anticancer activity, and wound healing. The search employed specific keywords such as “silver nanoparticles,” “antimicrobial activity,” “anticancer activity,” and “wound healing” to ensure thorough retrieval of relevant literature. Careful inclusion and exclusion criteria were applied to refine the dataset, resulting in a curated selection of publications for analysis. The initial data curation involved using Zotero version 6, a reference management software, to eliminate duplicates and effectively organize the literature (as per PRISMA guidelines in Figure 1). The curated dataset was then analyzed using the Bibliometrix R package, utilizing its Biblioshiny Version 4.1 interface for intuitive analysis (Aria and Cuccurullo, 2017). This bibliometric tool facilitated a comprehensive exploration of the dataset, extracting key metrics and trends within the field of study. For network visualization, VOSviewer version 1.6.20 was utilized to map relationships between key terms, authors, and publications, uncovering the intellectual structure and collaborative networks within the research domain. These visualizations provided insights into influential works, authors, and institutions contributing to the field. Additionally, Microsoft Excel 2019 was employed to create graphs and tables, presenting quantitative aspects of the dataset. This included the distribution of publications over time, citation analysis, and identification of core journals and articles shaping the discourse on AgNPs.

### 4.1 Data collection analysis

**FIGURE 2:**
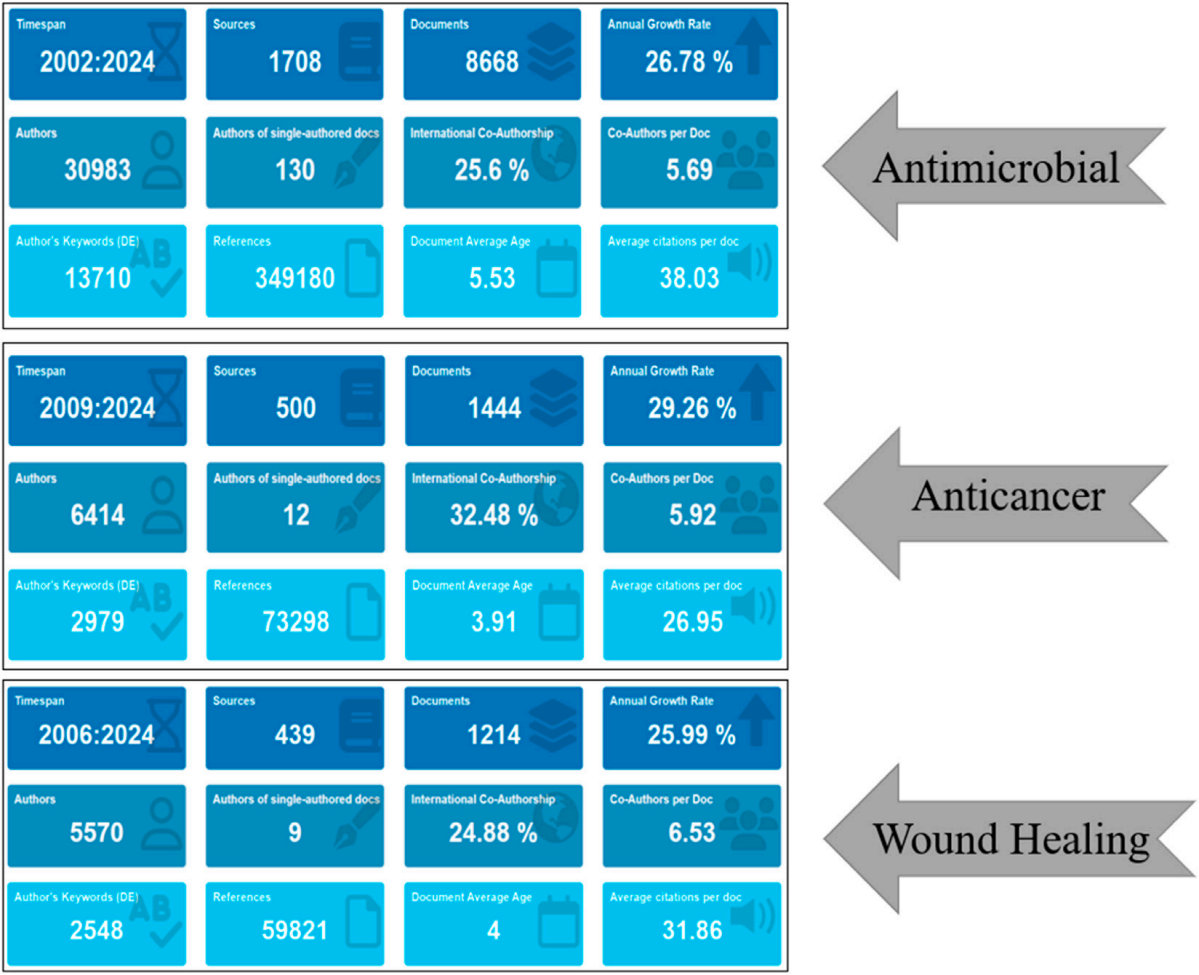
Comparative analysis of bibliometric data in silver nanoparticles research: a comprehensive overview delineating the dissemination and influence of scholarly works about silver nanoparticles, particularly emphasizing their antimicrobial, anticancer, and wound healing attributes throughout the periods of 2002–2024, 2009–2024, and 2006–2024 respectively. Vital metrics encompass the tally of sources, documents, authors, single-authored documents, percentage of international co-authorship, co-authors per document, author’s keywords, references, average age of documents, annual growth rate, and average citations per document.

The bibliometric examination of literature concerning silver nanoparticles reveals a thriving domain with noteworthy scientific contributions spanning three principal biomedical areas: antimicrobial, anticancer, and wound healing. The data (as shown in Figure 2) illustrates a substantial increase in publication volume over the specified periods, with antimicrobial applications leading with 8,668 documents (A), followed by wound healing with 1,214 documents (C), and anticancer research with 1,444 documents (B). This distribution underscores the prominence of silver nanoparticles in antimicrobial investigations and suggests a solid knowledge base supporting exploration into other medical domains (Naganthran et al., 2022).

Further analysis unveils intriguing dynamics in collaboration patterns and research depth. Antimicrobial research (A) not only exhibits the highest publication count but also demonstrates a notable average citation rate of 38.03, indicating the impactful nature of these studies. Additionally, there is a noteworthy international collaboration rate of 25.6%, underscoring global interest and cooperative efforts in combating microbial resistance. Similarly, anticancer (B) and wound healing (C) research show substantial international co-authorship at 32.48% and 24.88%, respectively, highlighting the global relevance and collaborative ethos within these research spheres. Remarkably, the average age of documents across all three fields is relatively low (ranging from 3.91 to 5.53 years), indicating an active and rapidly evolving field of research. The annual growth rates in these domains further emphasize the escalating interest and advancements, particularly notable in the anticancer domain (B) with a significant annual growth rate of 29.26%. This trend may reflect the pressing need for novel cancer therapies amidst global health challenges. Furthermore, the average number of co-authors per document indicates a collaborative trend in research endeavors, with wound healing investigations (C) exhibiting the highest at 6.53.

This implies that research in wound healing may necessitate interdisciplinary collaboration, drawing upon a variety of expertise to address the intricate mechanisms involved in tissue regeneration and infection management. In essence, the bibliometric data portrays a lively and cooperative research environment for silver nanoparticles, with notable contributions and interest spanning antimicrobial, anticancer, and wound healing investigations. This underscores the versatility of silver nanoparticles as a multifaceted tool in biomedicine, warranting ongoing exploration and investment. Additionally, the data emphasizes the importance of interdisciplinary approaches to fully leverage the potential of silver nanoparticles in tackling some of the most pressing health challenges of our era.

### 4.2 Yearly publication trend

**FIGURE 3:**
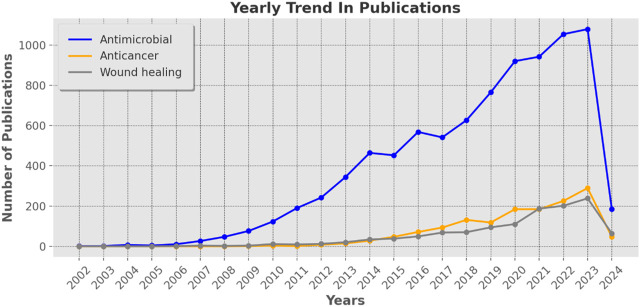
The graph depicts the annual progression of academic articles spanning from 2002 to 2024, showcasing the quantity of publications in three distinct research domains: antimicrobial, anticancer, and wound healing.

The examination of the annual publication trend in research concerning silver nanoparticles offers a descriptive narrative of the field’s progression and the increasing interest among researchers. (Bouafia et al., 2021). As depicted in the provided data Table 4, there has been a noticeable exponential surge in publication numbers across all three domains—antimicrobial, anticancer, and wound healing—over the past two decades. This trend is further visualized in the line graph (as shown in Figure 3), illustrating a particularly steep rise in publications related to antimicrobial properties, indicating a robust and sustained focus in this area.

**TABLE 4:** Yearly Publication Statistics for Silver Nanoparticles Research: This tabulated data provides the count of research papers published annually spanning from 2002 to 2024, categorized according to their exploration of the antimicrobial, anticancer, and wound healing attributes of silver nanoparticles.

Years	Antimicrobial	Anticancer	Wound healing
2002	1	-	-
2003	1	-	-
2004	7	-	-
2005	4	-	-
2006	10	-	1
2007	26	-	3
2008	47	-	2
2009	76	1	3
2010	123	3	11
2011	190	1	9
2012	242	8	12
2013	344	14	20
2014	464	28	34
2015	452	47	38
2016	568	71	49
2017	541	93	68
2018	627	131	70
2019	765	118	94
2020	920	184	110
2021	942	184	187
2022	1,054	225	201
2023	1,079	289	238
2024	185	47	64

The domain of antimicrobial research has witnessed a remarkable escalation from a single publication in 2002 to 1,079 in 2023, with a subsequent decline observed in 2024, possibly due to data collection cessation or a temporal anomaly requiring further investigation. Conversely, publications in the anticancer domain have demonstrated a more gradual yet consistent upward trajectory, reaching a noteworthy peak of 289 publications in 2023. Despite being the smallest in volume, research on wound healing exhibits a continuous increase in publications, highlighting an enduring and growing interest indicative of the importance placed on developing advanced wound management treatments. The upward trend in publications is not merely quantitative but also qualitative, evidenced by the escalating citations over time (as discussed in the previous section). This trend suggests that research is not only expanding in volume but also impact and relevance, with newer studies building upon and referencing previous work, contributing to a robust knowledge base. Such trends may be attributed to mounting evidence regarding the effectiveness of silver nanoparticles in these domains, coupled with technological advancements facilitating nanoscale research feasibility. Furthermore, the increase in interdisciplinary collaboration, reflected in the rising number of co-authors per document, likely played a pivotal role in advancing the field. In conclusion, the data illustrates an active and dynamically evolving field with a clear emphasis on the antimicrobial applications of silver nanoparticles, followed by substantial interest and significant research endeavors in anticancer and wound healing applications. These trends mirror the scientific community’s response to global health challenges and underscore the potential of nanotechnology to offer innovative solutions.

### 4.3 Publication distribution based on region

**FIGURE 4:**
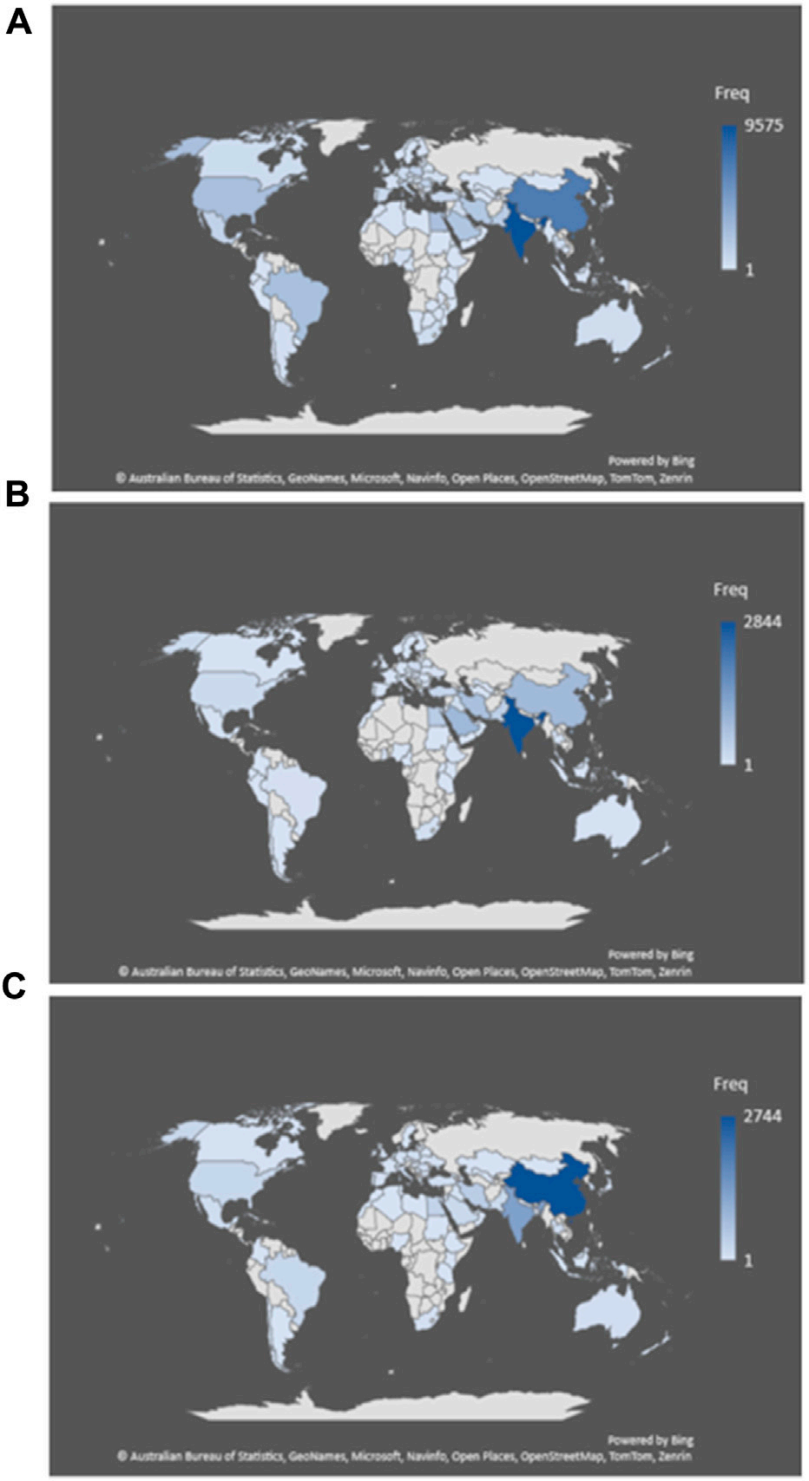
Global distribution of silver nanoparticles research publications: these maps present the frequency of research publications across different regions of the world for studies related to silver nanoparticles, categorized by their focus on antimicrobial **(A)**, anticancer **(B)**, and wound healing **(C)** properties.

The geographic analysis of publications concerning silver nanoparticle research (as depicted in Figure 4) unveils a notable discrepancy in research output across various regions, with specific countries demonstrating a concentrated presence (Li et al., 2022). Visualizing the data underscores a robust research footprint in India, China, and the United States, particularly in the realm of antimicrobial studies. This indicates a deliberate focus on utilizing silver nanoparticles for antimicrobial purposes in these regions, likely driven by the pressing need to address microbial resistance and infectious diseases, which are significant public health challenges.

Concerning anticancer research, the Indian subcontinent emerges as the primary contributor, with substantial frequencies also observed in Saudi Arabia, China, Iran, and South Korea. The investment in cancer research in these countries aligns with national health agendas aimed at innovating cancer treatment approaches using silver nanoparticles. In the domain of wound healing, China leads significantly, followed by India, Iran, Egypt, and Brazil. The distribution suggests a more geographically diverse interest in utilizing silver nanoparticles for wound healing, although it remains concentrated in countries with a specific focus on advanced wound care solutions. The heightened frequencies observed in these countries could be attributed to various factors, including the availability of research funding, the presence of robust scientific communities specializing in nanotechnology, and national healthcare priorities. Additionally, infrastructural capabilities for conducting advanced nanomaterial research may also influence this distribution. It is noteworthy that developing countries like India and China are prominently positioned across all three categories, indicative of their burgeoning research and development sectors. This underscores a global shift in scientific research hubs, with developing nations assuming more active roles in contributing to global knowledge, particularly in fields with significant societal implications like nanomedicine. The regional distribution underscores the importance of fostering international collaborations and knowledge exchange to bridge research disparities and harness the full potential of silver nanoparticles in healthcare. These insights are crucial for policymakers and funding agencies to comprehend the global research landscape and identify collaborative opportunities to advance the field of silver nanoparticles in medicine.

**FIGURE 5:**
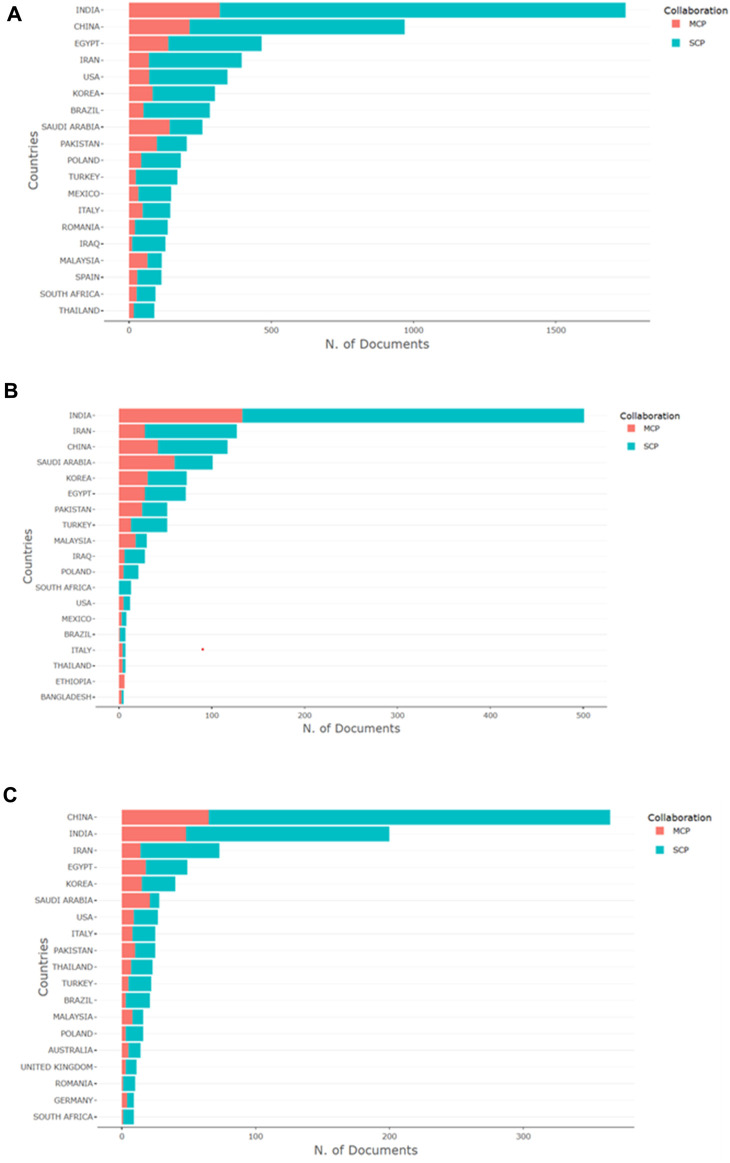
Collaborative patterns in silver nanoparticles research across nations: the figure illustrates the publication count from various countries, classified into single-country publications (SCP) and multiple-country publications (MCP), delineating collaboration trends in antimicrobial **(A)**, anticancer **(B)**, and wound healing **(C)** research involving silver nanoparticles.

The data depicted in Figure 5 clearly illustrates the collaborative endeavors in global silver nanoparticle research. The distinction between publications originating from a single country (SCP) and those involving multiple countries (MCP) offers valuable insights into international cooperation dynamics within this field (Syafiuddin et al., 2017). Across the antimicrobial, anticancer, and wound healing domains, SCPs dominate, indicating robust domestic research capabilities. However, the substantial representation of MCP highlights the concerted efforts of the global scientific community to tackle the multifaceted challenges associated with silver nanoparticles in these areas.

In the antimicrobial sector (A), India emerges with the highest document count, implying a potent synergy of local research initiatives and international alliances. The ratio of MCP to SCP suggests a willingness to engage in global collaborations, potentially fostering progress in this crucial domain. China follows a similar trajectory, balancing significant domestic research output with substantial international cooperation. Regarding anticancer research (B), while India leads in publication count, there’s a slight tilt towards SCP, hinting at a pronounced domestic focus on cancer research despite continued collaborative efforts. In the realm of wound healing (C), China takes the lead with a higher prevalence of SCP, possibly reflecting a strategic emphasis on localized solutions for wound management. Nonetheless, China also demonstrates substantial international collaboration, as evidenced by the presence of MCP.

The prevalence of MCP across all three fields underscores the international character of nanotechnology research, where the exchange of knowledge and resources plays a pivotal role in fostering innovation. It is noteworthy that developed nations like the USA, despite exhibiting lower overall publication counts in these charts, demonstrate a significant presence in MCP, indicating their substantial involvement in collaborative research beyond what mere publication figures might suggest. In essence, the data reflects a growing inclination toward global collaboration in silver nanoparticle research, essential for addressing the multifaceted challenges associated with their effective application in healthcare. Such collaborations offer numerous benefits, including the sharing of expertise, access to diverse research facilities, and a broad scientific perspective, all of which can accelerate the pace of discovery and application of silver nanoparticles across various medical domains.

### 4.4 Bibliometric analysis of authors, affiliations and journals

The bibliometric analysis of silver nanoparticle research offers valuable insights into the intellectual landscape of the field through a comprehensive examination of authors, affiliations, and journals. The data shown in Table 5 highlights a specific cohort of researchers who stand out due to their significant contributions. For instance, in the antimicrobial sector, ‘Wang Y’ and ‘Zhang Y’ emerge as leading contributors with 90 and 76 articles, respectively, demonstrating remarkable individual productivity that shapes discussions in this domain. Similar trends are observed in the fields of anticancer and wound healing, where individuals like ‘Guru Nathan S’ and ‘Wang Y’ have made substantial contributions, with 13 and 38 articles, respectively, showcasing their influential presence across multiple research areas.

**TABLE 5:** Key authors in research on silver nanoparticles: an overview of leading authors and their article counts in the domains of antimicrobial, anticancer, and wound healing research involving silver nanoparticles.

Silver nanoparticles domain					
Antimicrobial research		Anticancer research		Wound healing research	
Authors	Articles	Authors	Articles	Authors	Articles
Wang Y	90	Guru Nathan S	13	Wang Y	38
Zhang Y	76	Li Y	13	Liu X	27
Liu Y	66	Mortazavi-Derazkola S	13	Wang X	25
Li J	60	Nayaka S	13	Zhang Y	25
Liu X	59	Wang Y	13	Li Y	24
Li Y	57	Singh P	12	Zhang X	22
Zhang X	57	Zhang Y	11	Chen X	21
Wang X	54	Ebrahim Zadeh MA	10	Chen J	20
Wang L	51	Kumar V	10	Li X	20
Chen J	49	Yang DC	10	Liu Y	20

Scientific journals play a crucial role in sharing research findings, and publications such as the ‘International Journal of Nanomedicine’ and ‘The International Journal of Biological Macromolecules stand out with high article counts. This underscores their significance as primary platforms for the latest research on silver nanoparticles, highlighting their vital role in advancing the field and facilitating the exchange of knowledge (as depicted in Table 6).

**TABLE 6:** Top journals publishing research on silver nanoparticles: this table presents journals with the highest article counts across the antimicrobial, anticancer, and wound healing domains of silver nanoparticles research.

Silver nanoparticles domain					
Antimicrobial research		Anticancer research		Wound healing research	
Sources	Articles	Sources	Articles	Sources	Articles
International Journal of Biological Macromolecules	183	International Journal of Nanomedicine	44	International Journal of Biological Macromolecules	100
International Journal of Nanomedicine	115	Journal of Drug Delivery Science and Technology	27	Materials Science and Engineering C	31
Colloids and Surfaces B: Bio interfaces	108	Artificial Cells, Nanomedicine and Biotechnology	26	Carbohydrates Polymers	30
Nanomaterials	106	International Journal of Biologicals Macromolecules	24	ACS Applied Materials and Interfaces	26
Materials Science and Engineering C	104	Journals of Phytochemistry and Photobiology B: Biology	24	International Journal of Nanomedicines	24
RSC Advances	97	Nanomaterials	24	Advanced Healthcare Materials	18
Carbohydrates polymers	91	Bionanosciences	23	International Journals of Pharmaceuticals	18
ACS Applied Materials and Interfaces	89	Scientific reports	22	Pharmaceutics	17
International Journals of Molecular Sciences	87	Molecules	21	Journals of Materials Chemistry B	16
Molecules	83	Materials Science and Engineering C	20	Colloids and Surfaces B: Bio interfaces	15

Institutional affiliations serve as indicators of the research capabilities and areas of focus of academic and research institutions globally (Table 7 presents institutional contributions to Silver Nanoparticles Research) (Zahoor et al., 2021) King Saud University emerges as a leading institution in both antimicrobial and anticancer research, underscoring its deliberate investment in silver nanoparticle research. Additionally, institutions like Sichuan University and Southwest University are notable for their emphasis on wound-healing research.

**TABLE 7:** Institutional contributions to silver nanoparticles research: this table showcases the significant affiliations and their scholarly productivity in the realms of antimicrobial, anticancer, and wound healing research involving silver nanoparticles.

Silver nanoparticles domain					
Antimicrobial research		Anticancer research		Wound healing research	
Affiliation	Articles	Affiliation	Articles	Affiliation	Articles
King Saud University	828	King Saud University	399	Sichuan University	132
Islamic Azad University	254	Islamic Azad University	161	Southwest university	94
Kyung Hee University	211	Bharathidasan University	101	Islamic Azad University	61
National Research Centre	210	Karnatak University	87	King Saud University	61
Southwest University	201	Kyung Hee University	85	International University	59
Sichuan University	199	Konkuk University	72	National Research Centre	56
University of Belgrade	187	King Khalid University	71	Central South University	55
King Khalid University	167	Mazandaran University of Medical Sciences	71	Third Military Medical University	54
Cairo University	162	Periyar University	67	Northwest A&F University	48
Al-Azhar University	152	Alagappa University	61	Northwestern Polytechnical University	44

The distribution of prolific authors and institutions, along with the focus on prominent journals, indicates both regional and global trends in research priorities. It suggests that certain regions may be allocating more resources to silver nanoparticle research or have developed specialized expertise in this field. This insight can be highly beneficial for new researchers seeking collaboration opportunities or institutions looking to establish their presence in this area of study. This bibliometric analysis offers a snapshot of the key contributors and platforms driving advancements in our understanding of silver nanoparticles. It underscores the importance of ongoing support and collaboration within the scientific community to further explore the potential of silver nanoparticles in addressing global health challenges across antimicrobial, anticancer, and wound healing applications.

### 4.5 Citation impact across research domains of silver nanoparticles

The bibliometric examination of citations within silver nanoparticle research domains sheds light on the most influential studies (as shown in Table 8) that have significantly impacted the field (Yeung et al., 2020). For instance, in the realm of antimicrobial research, the paper titled “Silver nanoparticles as an antimicrobial agent: a case study on *E. coli* as a model for Gram-negative bacteria” has garnered an impressive 7,645 citations, indicating its pivotal role in establishing the efficacy of silver nanoparticles against bacterial infections (Sondi and Salopek-Sondi, 2004). Similarly, the top-cited paper in the anticancer research domain, with 481 citations, delves into the broader medical applications of nanoparticles, showcasing their diverse potential beyond antimicrobial uses. In the domain of wound healing, the integration of silver nanoparticles into bacterial cellulose for antimicrobial wound dressing, with 1,210 citations, exemplifies the innovative application of silver nanoparticles in improving wound care. The substantial citation counts across these papers underscore the significance of silver nanoparticles in driving advancements in medical research and the widespread recognition of these findings within the scientific community.

**TABLE 8:** Top cited papers in silver nanoparticles research: this table presents the most highly cited research papers in the domains of antimicrobial, anticancer, and wound healing research related to silver nanoparticles.

Silver nanoparticles domain						
Antimicrobial research		Anticancer research		Wound healing research		Reference
Paper title	Citations	Paper title	Citations	Paper title	Citations	
Silver nanoparticles as antimicrobial agent: a case study on *E. coli* as a model for Gram-negative bacteria	7,645	Medical applications of nanoparticles in biological imaging, cell labeling, antimicrobial agents, and anticancer nano drugs	481	Impregnation of silver nanoparticles into bacterial cellulose for antimicrobial wound dressing	1,210	Sondi and Salopek-Sondi (2004), Maneerung et al. (2008), Singh and Nalwa (2011)
Antimicrobial effects of silver nanoparticles	6,156	*Origanum vulgare* mediated biosynthesis of silver nanoparticles for its antibacterial and anticancer activity	477	Emerging Strategies to Combat ESKAPE Pathogens in the Era of Antimicrobial Resistance	1,302	Kim et al. (2007), Sankar et al. (2013), Mulani et al. (2019)
Does the antibacterial activity of silver nanoparticles depend on the shape of the nanoparticle? A study of the Gram-negative bacterium *Escherichia coli*	5,189	Biogenic silver nanoparticles for cancer treatment: an experimental report	498	Topical delivery of silver nanoparticles promotes wound healing	1,137	Pal et al. (2007), Tian et al. (2007), Jeyaraj et al. (2013)
Cytotoxicity and Genotoxicity of Silver Nanoparticles in Human Cells	3,924	Selective cytotoxicity of green synthesized silver nanoparticles against the MCF-7 tumor cell line and their enhanced antioxidant and antimicrobial properties	439	Plant-inspired adhesive and tough hydrogel based on Ag-Lignin nanoparticles-triggered dynamic redox catechol chemistry	708	AshaRani et al. (2009), Khorrami et al. (2018), Gan et al. (2019)
Silver colloid nanoparticles: synthesis, characterization, and their antibacterial activity	2,971	Antimicrobial and anticancer activities of silver nanoparticles synthesized from the root hair extract of *Phoenix dactylifera*	354	Antiangiogenic properties of silver nanoparticles	634	Panacek et al. (2006), Gurunathan et al. (2009), Oves et al. (2018)
Applications of nanotechnology in water and wastewater treatment	2,591	Biosynthesis of silver nanoparticles: Elucidation of prospective mechanism and therapeutic potential	379	*In situ* synthesis of silver-nanoparticles/bacterial cellulose composites for slow-released antimicrobial wound dressing	473	Qu et al. (2013), Mittal et al. (2014), Wu et al. (2014)
Applications of nanotechnology in food packaging and food safety: barrier materials, antimicrobials and sensors	2,345	*Acalypha indica Linn:* Biogenic synthesis of silver and gold nanoparticles and their cytotoxic effects against MDA-MB-231, human breast cancer cells	337	Development of novel chitin/nanosilver composite scaffolds for wound dressing applications	478	Madhumathi et al. (2010), Duncan (2011), Krishnaraj et al. (2014)
Strain specificity in antimicrobial activity of silver and copper nanoparticles	2,299	Comparative assessment of the apoptotic potential of silver nanoparticles synthesized by *Bacillus tequilensis* and *Calocybe indica* in MDA-MB-231 human breast cancer cells: targeting p53 for anticancer therapy	362	Novel Asymmetric Wettable AgNPs/Chitosan Wound Dressing: *In Vitro* and *In Vivo* Evaluation	351	Ruparelia et al. (2008), Gurunathan et al. (2015), Liang et al. (2016)
Negligible particle-specific antibacterial activity of silver nanoparticles	2,296	Green synthesis of silver nanoparticles using *Ganoderma neo-japonicum Imazeki*: a potential cytotoxic agent against breast cancer cells	343	Silver nanoparticle-impregnated chitosan-PEG hydrogel enhances wound healing in diabetes-induced rabbits	336	Xiu et al. (2012), Gurunathan et al. (2013), Masood et al. (2019)
Antibacterial activity and mechanism of silver nanoparticles on *Escherichia coli*	1927	Biosynthesis of Silver Nanoparticles Using *Cucumis prophetarum* Aqueous Leaf Extract and Their Antibacterial and Antiproliferative Activity Against Cancer Cell Lines	123	Preparation and characterization of novel β-chitin/nanosilver composite scaffolds for wound dressing applications	311	Kumar et al. (2010), Li et al. (2010), Hemlata et al. (2020)

**FIGURE 6:**
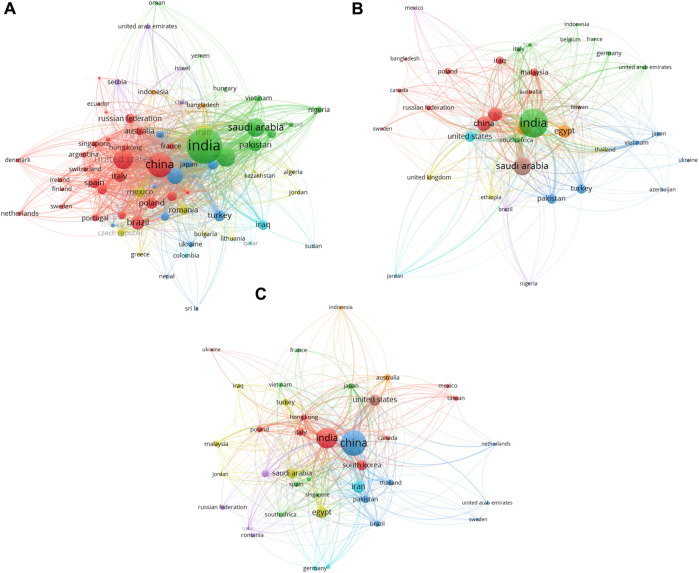
**(A)** Network visualizations of silver nanoparticles research: these visuals illustrate the bibliometric network encompassing research collaborations and citations in silver nanoparticle research across the antimicrobial domain. **(B)** Network visualizations of silver nanoparticles research: these visuals illustrate the bibliometric network encompassing research collaborations and citations in silver nanoparticles research across the anticancer domain. **(C)** Network visualizations of silver nanoparticles research: these visuals illustrate the bibliometric network encompassing research collaborations and citations in silver nanoparticles research across the wound healing domain.

From a geographical standpoint (as shown in Table 9), India leads in total citations across the antimicrobial and anticancer research domains, indicating the significant impact of its research output. Conversely, China has made considerable strides in the wound healing domain, accumulating the highest number of citations, underscoring its leading role in this area of research. The network visualization figures serve as graphical representations of the connections between countries and the collaborative nature of silver nanoparticle research (as shown in Figure 6). The size of the nodes (countries) and the thickness of the lines (collaborations) illustrate the extent of research activities and partnerships. These visualizations offer an intuitive grasp of how knowledge in the field is interconnected and how different regions contribute to and exchange information within the global research landscape. These bibliometric indicators—such as highly cited papers, total citations by country, and network visualizations—not only depict the current status of silver nanoparticle research but also inform future collaborations, funding decisions, and strategic priorities in the field. They underscore the importance of both individual scholarly works and collective international endeavors in advancing the understanding and application of silver nanoparticles for public health.

**TABLE 9:** Citation influence across countries in silver nanoparticles research: this table presents the cumulative citations garnered by research publications originating from different countries across the antimicrobial, anticancer, and wound healing spheres of silver nanoparticles.

Silver nanoparticles domain					
Antimicrobial research		Anticancer research		Wound healing research	
Country	Total citations	Country	Total citations	Country	Total citations
India	64,785	India	12,878	China	13,197
China	36,447	Korea	3,976	India	6,125
USA	29,079	Iran	3,692	Iran	1740
Korea	28,109	China	2,944	Egypt	1,584
Iran	11,754	Saudi Arabia	2,941	USA	1,534
Egypt	11,422	Egypt	1,393	Korea	1,269
Brazil	6,831	Pakistan	1,048	Hong Kong	1,179
Saudi Arabia	6,673	Malaysia	1,037	Italy	1,171
Singapore	6,004	Turkey	803	Thailand	1,157
United Kingdom	5,527	Iraq	455	Australia	722

### 4.6 Bibliometric analysis trends topics in silver nanoparticles research

**FIGURE 7:**
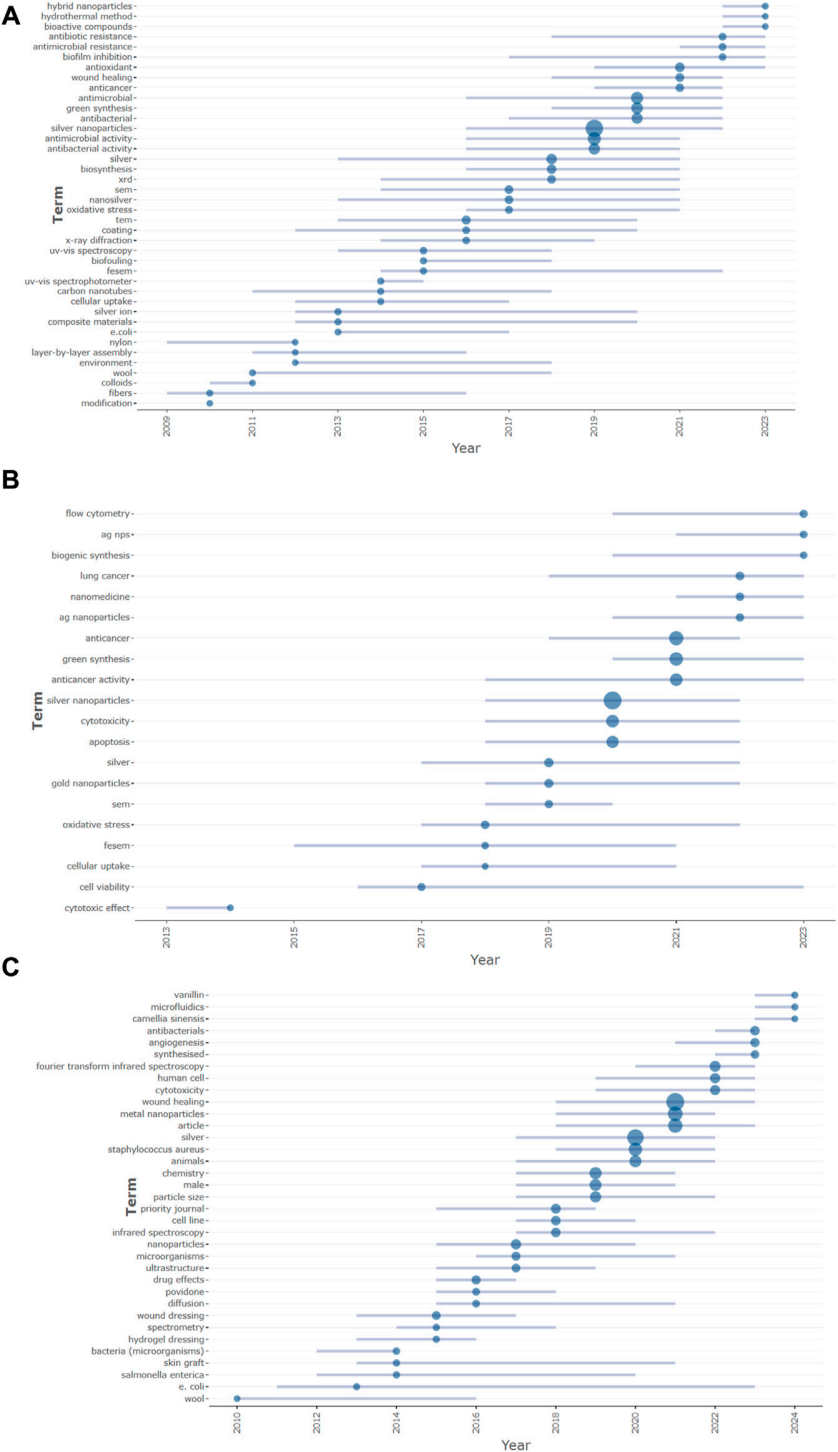
**(A)** Emerging themes in silver nanoparticles research: the figure comprises three horizon charts illustrating the occurrence and evolution of significant research themes over time in the realms of antimicrobial concerning silver nanoparticles, spanning from 2009 to 2024. **(B)** Emerging themes in silver nanoparticles research: the figure comprises three horizon charts illustrating the occurrence and evolution of significant research themes over time in the realms of anticancer concerning silver nanoparticles, spanning from 2009 to 2024. **(C)** Emerging themes in silver nanoparticles research: the figure comprises three horizon charts illustrating the occurrence and evolution of significant research themes over time in the realms of wound healing concerning silver nanoparticles, spanning from 2009 to 2024.

The bibliometric examination of emerging themes offers a comprehensive insight into the evolving research focus within silver nanoparticles research across three key domains: antimicrobial, anticancer, and wound healing (as shown in Figure 7) (Peng et al., 2023). In the antimicrobial domain (A), the prevalence of terms like “biofilm inhibition” and “antibiotic resistance” reflects a global health priority to combat antibiotic-resistant strains. The consistent occurrence of “antibacterial activity” underscores ongoing efforts to comprehend and enhance the antimicrobial effectiveness of silver nanoparticles. The emergence of “green synthesis” signifies a growing interest in environmentally friendly production methods, indicating a shift towards sustainable research practices.

In anticancer research (B), the prominence of terms such as “cytotoxicity” and “apoptosis” indicates a focused exploration of the mechanisms through which silver nanoparticles induce cancer cell death, crucial for the development of potential nanotherapeutics. The increasing prevalence of “nanomedicine” reflects the integration of silver nanoparticles into broader therapeutic contexts and the exploration of their role within multifunctional drug delivery systems.

In the wound healing domain (C), the term “hydrogel dressing” gaining traction suggests a significant interest in novel wound care materials incorporating silver nanoparticles. The repeated mention of “wound dressing” and “skin graft” underscores the potential of silver nanoparticles in promoting tissue regeneration and repair. The presence of “antibacterial” within wound healing emphasizes the dual functionality of silver nanoparticles, combining healing promotion with essential infection control.

These trends indicate a maturation of the field, transitioning from broad investigations into the antibacterial properties of silver nanoparticles towards specialized applications addressing specific challenges like resistant bacterial strains, targeted cancer therapy, and advanced wound care solutions. The size and clustering of terms within the horizon charts not only demonstrate the significance and relevance of these topics but also highlight the collaborative and interdisciplinary efforts propelling innovation in nanotechnology (Husain et al., 2023). In conclusion, this meticulous bibliometric analysis of emerging themes offers strategic insights into the development and potential future trajectories of silver nanoparticle research. It underscores the imperative for continued focus on sustainable production methods, targeted therapeutic applications, and the development of multifunctional materials to fully harness the benefits of silver nanoparticles in addressing critical healthcare challenges.

### 4.7 Thematic mapping

**FIGURE 8:**
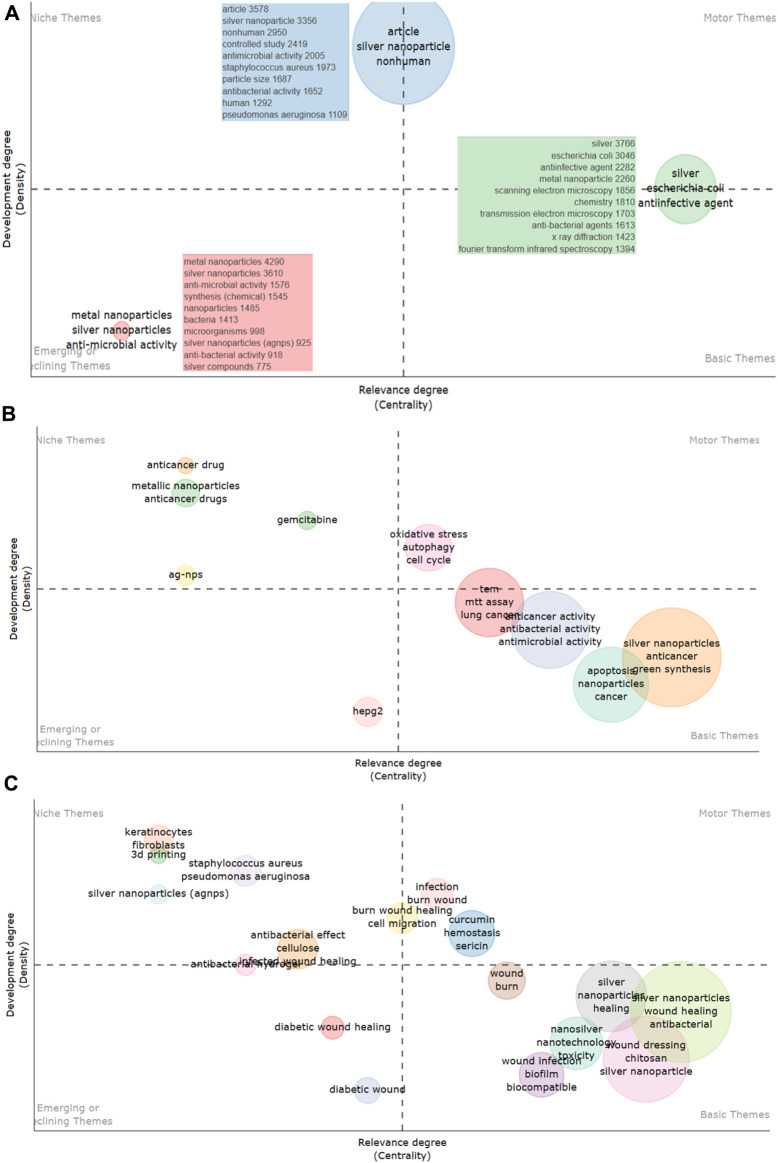
**(A)** Thematic mapping of silver nanoparticles research: these visual representations showcase the strategic layout of research themes on silver nanoparticles’ antimicrobial domain. Each map delineates niche, emerging, motor, and basic themes, discerning their centrality and density within the scholarly discourse on silver nanoparticles. **(B)** Thematic mapping of silver nanoparticles research: these visual representations showcase the strategic layout of research themes on silver nanoparticles anticancer domain. Each map delineates niche, emerging, motor, and basic themes, discerning their centrality and density within the scholarly discourse on silver nanoparticles. **(C)** Thematic mapping of silver nanoparticles research: these visual representations showcase the strategic layout of research themes on silver nanoparticles wound healing domain. Each map delineates niche, emerging, motor, and basic themes, discerning their centrality and density within the scholarly discourse on silver nanoparticles.

The thematic maps provided here offer a visual breakdown of the silver nanoparticles research landscape, categorizing themes into niche, emerging, motor, and basic categories based on their centrality (importance) and density (advancement) in the literature (as shown in Figure 8) (Di Cosmo et al., 2021).

In the antimicrobial domain (A), terms like “silver nanoparticles,” “antibacterial activity,” and “antimicrobial” are classified as basic themes, indicating well-established, fundamental research areas with high centrality and development. Motor themes such as “*E. coli”* and “biofilm” represent highly developed and central areas that drive research in the field. Niche themes like “controlled study” and “silver nanocomposite” have lower centrality but are highly developed, signifying specialized yet significant areas of study. Emerging or declining themes, such as “silver compounds” and “biosynthesis,” show lower density but are gaining or losing centrality, indicating evolving areas of interest.

In the anticancer domain (B), central clusters around “silver nanoparticles,” “anticancer activity,” and “green synthesis” demonstrate a strong foundational presence, indicating extensive study and centrality in the field. Motor themes like “gemcitabine” and “oxidative stress” highlight influential topics driving research. Niche themes such as “metallic nanoparticles” indicate high development but lower centrality, representing specialized areas. Emerging themes like “ag-nps” and “hepG2” suggest nascent research areas with growing centrality.

➢ For the wound healing domain (C), basic themes like “silver nanoparticles” and “antibacterial” form the core of research, showing high centrality and development. Motor themes such as “nanotechnology” and “wound dressing” are central to driving the field and are well-developed. Niche themes like “keratinocytes” and “3D printing” are highly developed but with lower centrality, indicating specialization yet impact. Emerging themes like “diabetic wound healing” have started to gain centrality, indicating new frontiers in the field. Thematic mapping serves as a strategic tool for identifying research focus and trajectory. In the context of silver nanoparticles, these maps provide a clear framework of mature and developing topics, offering insights into areas ripe for exploration and investment. The persistence of certain terms reflects the enduring importance of foundational research, while the emergence of new themes highlights shifts in scientific inquiry and potential applications for silver nanoparticles in these domains.

### 4.8 Discussion

The bibliometric analysis of the scientific literature on silver nanoparticles (AgNPs) shows how the field has grown and developed. In this section, we discuss the implications that these results have: the general impact on the scientific community, potential applications, and future research directions.

➢ Strategic Focus on Antimicrobial Properties: Our findings indicate that AgNPs exert a massive effect in combating the global health crisis caused by antimicrobial resistance. A considerable amount of literature and citations in this field is a clear sign of not only the relevance of AgNPs as potent antimicrobial agents but also a call for finding alternatives to traditional antibiotics. The fact that there is ongoing research along this line provides good evidence that investments will pay off with significant breakthroughs in infectious disease management (Mohanta and Behera, 2014; Mohanta et al., 2017).
➢ Research on the antimicrobial activity of AgNPs continues to dominate; however, there is significant growth in research related to their anticancer and wound-healing potential. The growing research reflects the promising capability of AgNPs for future use in designing targeted therapeutic agents offering more localized therapy, thus minimizing the side effects of conventional cancer therapies. In wound healing, the promise of AgNPs in advanced dressings lies in their effectiveness at warding off infections and accelerating tissue regeneration. All these tendencies hint at increasing awareness of the multifunctionality of AgNPs, which might open the door to the revision of treatment protocols not only in oncology but also in dermatology (Mohanta et al., 2023; Mohanta et al., 2020).
➢ Role of International Collaboration: The present analysis demonstrates that international collaboration is focused intensively on the study of AgNPs worldwide. The present strong emphasis on interdisciplinary research, particularly in wound-healing-related studies, therefore necessitates harnessing the inherent properties of AgNPs from a consolidated information base of diverse expertise, resources, and technological innovations to quicken the pace of discovery and application.
➢ Regional Contributions and Future Directions: India with its enormous talent pool and cost-effective research environment, and China are both making tremendous contributions to furthering innovation in the AgNP research scenario. The focused research output from these regions has shown their commitment to addressing local and global health challenges and placed them at the forefront of nanomedicine research contributors. In future research, work on the long-term consequences of using AgNPs should focus more on their toxicity and environmental effects. Moreover, some areas, including the use of AgNPs in neurology and their interaction with the human microbiome, remain untapped for their potential to increase the applications of these nanoparticles.
➢ Policy Implications: International research collaboration: The distribution of scientific efforts over an extensive geographical area and the evident impact that international cooperation can have indicate the need to promote policies that facilitate collaborative research and innovation in nanotechnology. A framework, therefore, must be developed by policymakers to enhance international partnerships, while funding models are incentivized to advocate for research into sustainable and safe applications in nanotechnologies. This discussion not only places the relevant findings of this bibliometric analysis within context but also maps a potential trajectory for future research and development in silver nanoparticles, pointing out the critical domains that need further exploration and the strategic role of international collaboration in fostering advancements (Peng et al., 2023).
➢ Potential limitations of AgNPs in biomedical applications include concerns about their long-term toxicity and potential accumulation in tissues, the development of bacterial resistance, and the challenge of ensuring precise targeting and controlled release in drug delivery systems. Additionally, the variability in AgNP synthesis methods and characterization techniques can lead to inconsistencies in their properties and performance, posing challenges for standardized clinical use (Saddik et al., 2024) (130).

## 5 Translational research and clinical applications of silver nanoparticles in antimicrobial, anticancer, and wound healing treatments

Recent translational research has demonstrated the significant potential of silver nanoparticles (AgNPs) in various biomedical applications. In antimicrobial applications, AgNPs have shown enhanced efficacy in wound healing by promoting fibroblast migration and reducing infection rates. For instance, silver nanomix has been developed as an effective wound dressing material, showing superior antibacterial activity compared to traditional silver products (Polaka and Tekade, 2023). In cancer treatment, AgNPs synthesized from *Bacillus sp*. KFU36 has demonstrated significant anticancer effects in breast cancer cells by inducing apoptosis (Almalki and Khalifa, 2020). Moreover, AgNPs combined with antimicrobial peptides and blue light have shown promising results in treating methicillin-resistant *S. aureus* (MRSA) infections, accelerating wound healing in diabetic rats (Ibrahim et al., 2019). FDA-approved products like Acticoat (Smith and Nephew) for wound dressings, Silvadene (Pfizer) for burn treatment, and Cytolux (Nanobiotix) for targeted cancer therapy exemplify the clinical translation of AgNP-based therapies. Additionally, studies have highlighted the use of AgNPs in chitosan-based hydrogels, demonstrating enhanced wound-healing capabilities in diabetic rabbits (Masood et al., 2019). These products, along with ongoing clinical trials, underscore the translational potential and therapeutic efficacy of AgNPs in antimicrobial, anticancer, and wound healing treatments (as mentioned in Table 10).

**TABLE 10:** Overview of FDA-Approved products for silver nanoparticles in antimicrobial, anticancer, and wound healing applications.

Product name	Application	Key clinical information	Manufacturer	Regulatory approval	Approval year
Acticoat	Wound Healing	Effective against over 150 pathogens, applied every 3–7 days	Smith and Nephew	FDA	2002
SilvaSorb	Wound Healing	Sustained release for up to 7 days, reduces bioburden	Medline Industries	FDA	2003
Silverlon	Wound Healing	Enhances healing, reduces infection, effective for 3 days	Argentum Medical	FDA	2004
SilvaGard	Antimicrobial	Applied to medical devices, provides long-term antimicrobial protection	NanoHorizons	FDA	2005
SilverSeal	Wound Healing	Non-adherent, antimicrobial protection for up to 7 days	Encompass Group	FDA	2006
SilverStream	Antimicrobial	Wound irrigation solution reduces microbial load, used daily	Enzyme Solutions	FDA	2007
AgActive	Antimicrobial	Additives for various products, proven antimicrobial efficacy	BioCote	EU	2014
Nanosilver	Anticancer	Nanoparticle-based therapy targets tumor cells, and dosage varies by protocol	CytImmune Sciences	FDA	2015
Silvercel	Wound Healing	Alginate dressing, effective for up to 7 days, absorbs exudate	Systagenix	FDA	2008
SilverStat	Wound Healing	The antimicrobial gel promotes healing, applied twice daily	Nutralegacy	FDA	2010
SilverSorb	Wound Healing	Antimicrobial dressing, provides moisture balance, effective for up to 7 days	Medline Industries	FDA	2011
SilvaDerm	Wound Healing	Wound gel provides antimicrobial protection, and is effective for up to 7 days	Smith and Nephew	FDA	2016
SilverActive	Antimicrobial	Wound spray reduces infection, applied daily	Active Medical Products	EU	2016
NanoShield	Antimicrobial	Surface coating, long-lasting protection, reapply as needed	NanoLabs	EU	2017
SilverArmor	Antimicrobial	Advanced wound dressing, effective for 5–7 days	Medline Industries	FDA	2023

## 6 Challenges and future prospects in silver nanoparticles research: a SWOT perspective

As the scientific community delves deeper into the realm of silver nanoparticles (AgNPs), it becomes imperative to strategically assess both the challenges and opportunities inherent in this innovative field. The ensuing SWOT analysis serves as a crucial tool in dissecting the complexities and predicting the trajectory of AgNP research (Dhir et al., 2024). Challenges in this domain are multifaceted, with concerns such as potential cytotoxicity and the development of resistance taking precedence in scientific discourse. The economic viability of producing AgNPs on a large scale, compounded by a lack of comprehensive long-term safety data, further complicates their clinical and commercial applications. Additionally, the evolving regulatory landscape and environmental ramifications of nanoparticle utilization necessitate thorough scrutiny and the adoption of sustainable practices. However, the future holds considerable promise for AgNPs, driven by the growing demand for advanced antimicrobial and anticancer therapies in healthcare. Advances in nanotechnology bolster the prospects for AgNPs, offering innovative applications through targeted drug delivery systems and synergistic therapeutic modalities. The versatility of AgNPs in integrating with various platforms indicates their expanding role in medical interventions (Tunç, 2024; Rameshkumar et al., 2023).

**FIGURE 9:**
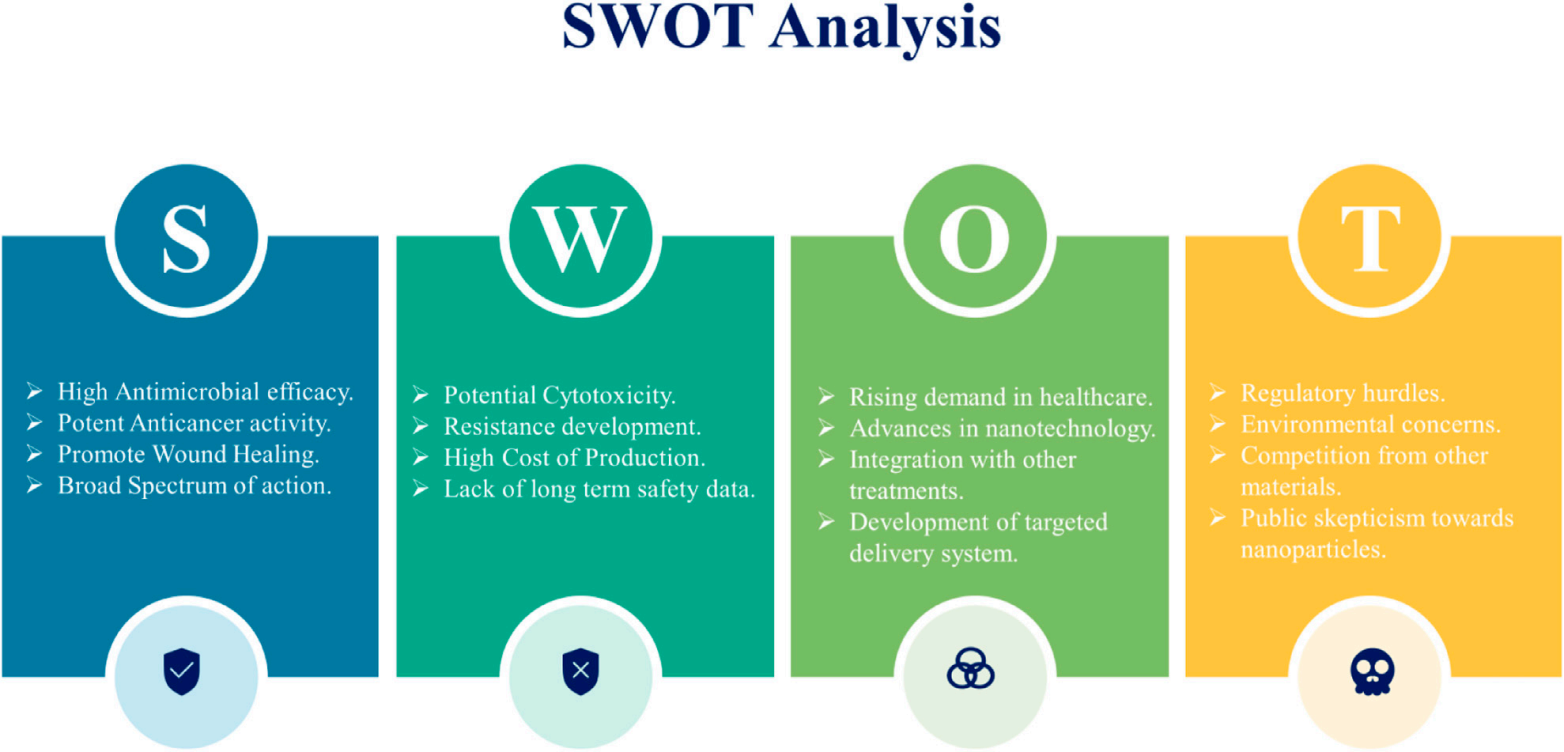
Strategic evaluation of silver nanoparticles research: A SWOT analysis (Khuda et al., 2023).

The SWOT analysis embedded within this context provides a holistic overview of the internal and external factors influencing the field (as shown in Figure 9): (Hasan et al., 2022)

➢ Strengths such as the well-established antimicrobial and wound healing properties of AgNPs, coupled with their broad spectrum of action, reinforce their indispensable role in healthcare.
➢ Weaknesses, including the high production costs and concerns regarding cytotoxic effects, present tangible challenges that research and development efforts must address.
➢ Opportunities abound, with advancements in nanotechnology creating new pathways for the application of AgNPs across diverse biomedical domains.
➢ Threats are characterized by regulatory hurdles, environmental concerns, competition from alternative materials, and public apprehensions surrounding nanotechnology.

Recent studies have documented resistance to silver nanoparticles (AgNPs) as an emergent problem in their role as antimicrobial agents. Many studies have established that bacteria can develop resistance to AgNPs through different adaptive mechanisms. For example, one study by Panacek et al. (2017) has shown that bacteria like *E. coli* and *Pseudomonas aeruginosa* do so by producing flagellin that induces aggregation of the nanoparticles, thereby reducing their efficacy. Reduced interaction has been observed when AgNPs are exposed to pathogens that produce organic acids, resulting in microbial resistance (Panáček et al., 2018). In contrast, further research conducted by Lu (2019) showed that both silver ions and AgNPs can facilitate the horizontal transfer of plasmid-mediated antibiotic resistance genes, thus emphasizing the ecological risks associated with these materials in widespread use (Lu et al., 2020). Also, biofilm formation and enzymatic reduction of Ag^+^ to less toxic forms are other discovered mechanisms of resistance identified within bacterial strains, consequently reducing the effectiveness of silver as an antimicrobial agent (Kaweeteerawat et al., 2017; Ellis et al., 2018). In addition, AgNPs reported by Yuan (2017) further indicated that the biologically synthesized AgNPs exhibit potential toward MDR bacteria but acquire resistance (Yuan et al., 2017). Thus, there is a need to consider their judicious use in combination with novel methods that would prevent any resistance development. Along this line, the issues of bacterial resistance to silver nanoparticles remain equally sensitive, also bearing in mind the fact that in the long term, they would have to be used as antimicrobial agents, a process that will always be ongoing due to constant research being done to finding innovative ways. Together, this evaluation underscores the need for a nuanced approach to advancing AgNP research and highlights the importance of foresight in navigating potential obstacles. It advocates for a balanced approach that capitalizes on the strengths and opportunities of AgNPs while mitigating the weaknesses and threats. The forthcoming years are pivotal, demanding concerted efforts to overcome challenges and fully harness the transformative potential of silver nanoparticles in biomedical science and therapeutics (Beyene et al., 2017).

## 7 Conclusion

The comprehensive bibliometric analysis conducted in this review sheds light on the extensive and multifaceted expansion of silver nanoparticle research across the domains of antimicrobial, anticancer, and wound healing. The data underscore the crucial role of AgNPs as an invaluable asset in the biomedical field, addressing some of the most pressing health challenges of our time. In antimicrobial research, AgNPs have demonstrated their critical ability to combat drug-resistant bacteria, highlighting their potential to revolutionize infectious disease treatment. In oncology, AgNPs show promise as anticancer agents, opening new avenues in cancer therapy by inducing targeted cytotoxicity. Additionally, research in wound healing reveals the effectiveness of AgNPs in promoting tissue regeneration and preventing infections, representing a significant advancement in medical treatments. The geographical analysis of research output indicates a shift towards a more globally distributed contribution, with countries like India and China leading in both publication and citations, suggesting a growing decentralization of research activity. The rise in topics such as green synthesis methods reflects a conscious effort towards sustainable practices in nanoparticle production. Future perspectives derived from this analysis suggest a continued trajectory of growth and innovation, with interdisciplinary collaborations and sustainable development taking center stage. It is expected that AgNPs will continue to drive nanotechnological advancements, significantly contributing to the development of next-generation medical treatments. As the research community delves further into the unexplored applications of AgNPs, it is crucial to consider not only their scientific and therapeutic potential but also their environmental and ethical implications.

## Data Availability

The raw data supporting the conclusions of this article will be made available by the authors, without undue reservation.
